# High blood viscosity in acute ischemic stroke

**DOI:** 10.3389/fneur.2023.1320773

**Published:** 2023-11-30

**Authors:** Jihoon Kang, Ju Seok Oh, Beom Joon Kim, Jun Yup Kim, Do Yeon Kim, So-Yeon Yun, Moon-Ku Han, Hee-Joon Bae, Inwon Park, Jae Hyuk Lee, You Hwan Jo, Kyung Hyun Ahn

**Affiliations:** ^1^Department of Neurology, Cerebrovascular Center, Seoul National University Bundang Hospital, Seongnam, Republic of Korea; ^2^Department of Advanced Materials and Chemical Engineering, Hannam University, Daejeon, Republic of Korea; ^3^Department of Emergency Medicine, Seoul National University Bundang Hospital, Seongnam, Republic of Korea; ^4^School of Chemical and Biological Engineering, Institute of Chemical Processes, Seoul National University, Seoul, Republic of Korea

**Keywords:** blood, viscosity, parallel plate, ischemic stroke, acute stroke

## Abstract

**Background:**

The changes in blood viscosity can influence the shear stress at the vessel wall, but there is limited evidence regarding the impact on thrombogenesis and acute stroke. We aimed to investigate the effect of blood viscosity on stroke and the clinical utility of blood viscosity measurements obtained immediately upon hospital arrival.

**Methods:**

Patients with suspected stroke visiting the hospital within 24 h of the last known well time were enrolled. Point-of-care testing was used to obtain blood viscosity measurements before intravenous fluid infusion. Blood viscosity was measured as the reactive torque generated at three oscillatory frequencies (1, 5, and 10 rad/sec). Blood viscosity results were compared among patients with ischemic stroke, hemorrhagic stroke, and stroke mimics diagnosed as other than stroke.

**Results:**

Among 112 enrolled patients, blood viscosity measurements were accomplished within 2.4 ± 1.3 min of vessel puncture. At an oscillatory frequency of 10 rad/sec, blood viscosity differed significantly between the ischemic stroke (24.2 ± 4.9 *centipoise, cP*) and stroke mimic groups (17.8 ± 6.5 *cP*, *p* < 0.001). This finding was consistent at different oscillatory frequencies (134.2 ± 46.3 vs. 102.4 ± 47.2 at 1 rad/sec and 39.2 ± 11.5 vs. 30.4 ± 12.4 at 5 rad/sec, Ps < 0.001), suggesting a relationship between decreases in viscosity and shear rate. The area under the receiver operating curve for differentiating cases of stroke from stroke mimic was 0.79 (95% confidence interval, 0.69–0.88).

**Conclusion:**

Patients with ischemic stroke exhibit increases in whole blood viscosity, suggesting that blood viscosity measurements can aid in differentiating ischemic stroke from other diseases.

## 1 Introduction

Hemostatic balance of blood circulation is essential for maintaining physiological conditions, even in those with risk factors such as atrial fibrillation, high blood pressure, and rupture-prone atherosclerosis (1). When physiologic homeostasis breaks down by some instance, it activates pro-coagulatory pathways and intermittently leads to adverse thrombogenic events, such as ischemic stroke. (1, 2).

Endothelial damage of blood vessels is pivotal in initiating such a prothrombotic state (3). In normal conditions, the blood circulates consistently by imposing a force on the vascular wall; however, when this force exceeds a threshold, damage to the endothelium or pre-existing plaque’s fibrous cap can occur, promoting subsequent pro-coagulation processes (4, 5). From the perspective of hemodynamics, the inertial force by blood flow to the vascular wall is measured with shear stress. Physiologically, this shear stress is proportional to the velocity of blood flow and blood viscosity, and their changes make the difference in the force on the endothelium (6). Investigations encountered that the velocity of flow, that is, shear rate, is measured at 1,000 s-1 in normal arteries, but it can be increased to over 5,000 s-1 in some forms of pathologic atherosclerosis (7). There were supporting pieces of evidence that such high shear rates exacerbate the progression of atherosclerosis and substantially increase the risk of rupture (8, 9).

However, as the shear rate effect is given to the long-standing, even in stable, it alone cannot adequately explain the rapid differential change of physical shear stress in the vasculature. To complement, it needs to draw attention to the blood viscosity, another proportional determinant of the shear stress level, which may also account for fluctuating conditions caused by heterogeneous components involved in biological processes, such as hematocrit, deformity of RBC, fibrinogen, and lipoprotein (10, 11). In addition, there is limited evidence to date regarding the role of high blood viscosity in acute thrombogenic events, especially stroke (12–14).

In the present study, we aimed to investigate the effect of blood viscosity on stroke occurrence and the clinical utility of blood viscosity measurements obtained immediately upon hospital arrival. To achieve this aim, we targeted the acute stroke suspects and compared blood viscosity measurements according to their final diagnosis between stroke and other diseases, namely, stroke mimics. A parallel plate rheometer designed for point-of-care testing (POCT) allows real-time measurement after blood sampling, where it is recommended to proceed within several minutes of hospital arrival before giving any drugs or treatments (15). This method is particularly advantageous in ensuring more accurate measurements of pure blood viscosity because it can avoid using any additional anticoagulant such as ethylenediaminetetraacetic acid (12, 16).

## 2 Methods

### 2.1 Study population and data collection

Patients who visited the emergency department with focal neurological symptoms at least 24 h after the last known well time were enrolled. At the triage, patients suspected of having a stroke are classified differentially within 5 min of arrival, and a critical pathway is activated, which enables them to contact the neurological team in the next 10 min. The sudden onset of asymmetrical weakness, sensory change, language disturbance, or any other new neurologic symptoms, such as change of mental status, dizziness, and visual disturbance, were suspected. With the proceeding of the rapid neurological assessment, it secures venous puncture for laboratory tests and intravenous catheter (Supplementary Figure S1). For this study, informed consent to participate and additional blood samples were obtained at this time and occurred within approximately 15 min by a practice-independent researcher. Blood sampling was performed before administering intravenous fluid or medication to avoid the influence of external factors on viscosity measurements. Cases in which blood sampling was deficient or delayed due to vascular fragility or low blood volume were excluded.

For all enrolled patients, diagnoses were confirmed by blinded neurologists based on medical information and imaging studies after discharge, following which patients were classified into ischemic stroke, hemorrhagic stroke, and stroke mimic (or non-stroke) groups. In some cases, patients transferred due to ischemic stroke from other institutions were separately classified into an ischemic stroke with intravenous (IV) fluid group. The following clinical data were also collected for all patients: demographic information; cardiovascular risk factors including hypertension, diabetes mellitus (DM), and dyslipidemia; initial laboratory findings including white blood cell (WBC) count, hemoglobin, hematocrit, platelets, lipid battery results, C-reactive protein (CRP), D-dimer, fibrinogen, glucose, blood urea nitrogen (BUN), creatinine, and prothrombin time (PT).

### 2.2 Study protocol and consent

The local institutional review board of Seoul National University Bundang Hospital (Republic of Korea) approved this study. Patients or caregivers provided written informed consent for additional blood sampling and viscosity measurements. The use of registered data is permitted upon request after the review.

### 2.3 Estimation of blood viscosity

**Figure 1:**
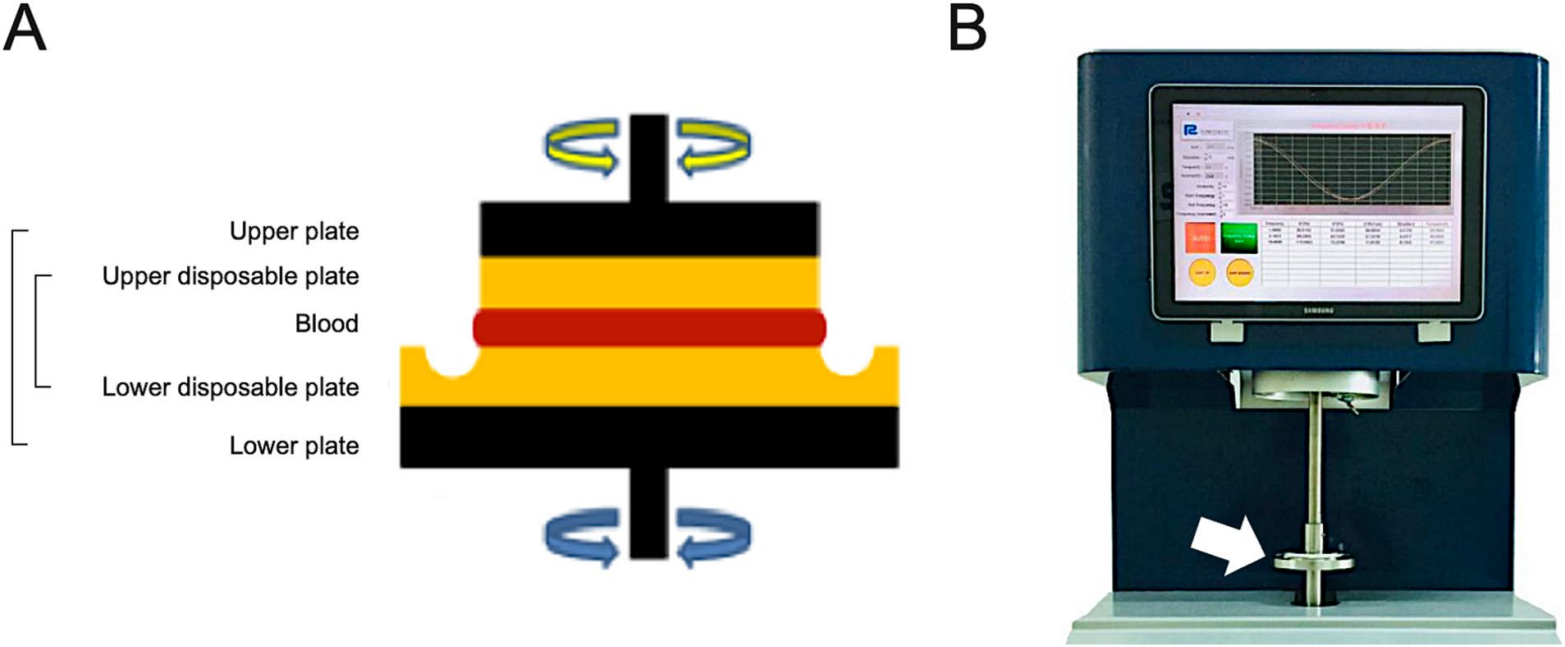
Schematic illustration of the core part measuring the blood viscosity of parallel plate rheometer **(A)** and the overall feature of ARS-Medi device **(B)**. After raising the upper metal plate (black color), a disposable plate was installed (yellow color). Blood samples were then infused into the disposable plate, and the upper metal plate was lowered in increments of 5 *mm* to adjust the rotational oscillatory frequency **(A)**. The device measured the reactive torque generated by the blood to estimate the blood viscosity.

Blood viscosity was measured using a parallel plate rheometer (ARS-Medi, Advanced Rheology Solutions, Ltd., Republic of Korea) designed for point-of-care and approved by the Korean Regional Food and Drug Administration. This method has been widely adopted for micro-scale structures and dispersed fluids as a fast and accurate measurement without data loss (Supplementary Table S1) (17, 18). A 2-*mL* whole blood sample was evenly distributed on the parallel plate (Figure 1). Testing was performed within 3 min of sampling and without anticoagulant reagents to ensure measurement of pure blood values. Oscillation frequencies were 1, 5, and 10 rad/sec, and the resultant values at each frequency were used to estimate the reactive torque from the blood sample.

### 2.4 Statistical analysis

Baseline characteristics, blood laboratory profiles, and blood viscosity of study population were summarized. The Pearson correlation test analyzed correlations between blood viscosity and various laboratory profiles.

The blood viscosity among diagnoses of ischemic stroke, ischemic stroke with IV fluid, hemorrhagic stroke, and stroke mimic were compared by the analysis of variance (ANOVA) test. Then, the ability of blood viscosity to differentiate between ischemic stroke and stroke mimics was tested using the area under the receiver operating curve (AUROC). All statistical analyses were conducted using SPSS (version 22.0, IBM) and R software (version 4.2.1).

## 3 Results

This study enrolled 112 patients with a mean age of 69.1 ± 13.3 years, 56.9% of whom were male, after excluding those with low blood sample volume (< 1 *mL*) and time delay during sampling. POCT measurements successfully obtained the blood viscosity within an average of 2.4 ± 1.3 min following a venular puncture. Blood viscosity values decreased with increasing the oscillation frequency, which was 115.4 ± 47.8 *cP*, 34.2 ± 12.2 *cP*, and 20.3 ± 6.5 *cP* at oscillatory frequencies of 1, 5, and 10 rad/sec, respectively (Supplementary Figure S2).

Blood viscosity exhibited significant correlations with hemoglobin (*γ* = 0.27, P by Pearson correlation test = 0.004) but not with WBC count (*γ* = 0.10, *p* = 0.52), hematocrit (*γ* = 0.23, *p* = 0.08). Among blood chemistry tests, total cholesterol and BUN were significantly correlated with blood viscosity (*γ* = 0.26, *p* = 0.01 and *γ* = −0.21, *p* = 0.03, Supplementary Table S2).

### 3.1 Disease groups and blood viscosity

The enrolled patients were classified into stroke mimics (n = 53), ischemic stroke (n = 53), and hemorrhagic stroke (n = 6, 5.4%). There was no difference in the distribution of age, hypertension, and diabetes mellitus; however, the proportion of dyslipidemia, CRP, and INR levels significantly differed according to the disease group (Table 1). The higher CRP values of the stroke mimic group were observed in systemic infection or encephalopathy (Supplementary Table S3).

**Table 1:** Baseline characteristics and diagnoses of enrolled patients (*n* = 112).

Variables	Stroke mimic (*n* = 53)	Ischemic stroke (*n* = 42)	Hemorrhagic stroke (*n* = 6)	*p*
Age, mean ± SD	67.1 ± 14.2	70.0 ± 12.4	70.0 ± 13.8	0.44
Male	28 (52.8%)	36 (67.9%)	1 (16.7%)	0.03
Hypertension	23 (43.4%)	31 (58.5%)	2 (33.3%)	0.21
Diabetes mellitus	15 (28.3%)	17 (32.1%)	2 (33.3%)	0.90
Dyslipidemia	6 (11.3%)	26 (49.1%)	1 (16.7%)	< 0.001
WBC (103/μL)	9.2 ± 6.6	6.9 ± 2.0	13.1 ± 11.6	0.93
Hemoglobin (g/dL)	13.3 ± 2.5	14.0 ± 2.0	14.4 ± 1.9	0.18
Hematocrit (%)	39.8 ± 6.9	42.1 ± 5.4	42.8 ± 5.3	0.12
Platelet (103/μL)	224.7 ± 84.1	224.0 ± 64.6	265.3 ± 102.0	0.45
Fibrinogen (mg/dL)	392.6 ± 162.2	325.2 ± 72.7	330.5 ± 122.2	0.03
D-dimer (g/L)	1.7 ± 1.6	0.9 ± 1.1	0.5 ± 0.01	0.22
CRP (mg/L)	3.8 ± 7.7	0.5 ± 0.8	1.1 ± 1.1	0.03
Glucose (mg/dL)	132.9 ± 51.9	131.3 ± 42.7	138.3 ± 39.2	0.90
Total cholesterol (mg/dL)	159.9 ± 47.7	174.6 ± 45.4	197.5 ± 76.3	0.17
HDL cholesterol (mg/dL)	49.2 ± 16.0	50.1 ± 11.9	62.7 ± 22.2	0.21
LDL cholesterol (mg/dL)	91.7 ± 33.1	107.4 ± 40.9	121.7 ± 54.4	0.10
BUN (mg/dL)	26.2 ± 29.4	17.0 ± 6.7	23.2 ± 15.4	0.17
Creatinine (mg/dL)	2.8 ± 1.2	0.9 ± 0.3	1.0 ± 1.0	0.72
INR	1.0 ± 0.1	1.0 ± 0.1	1.5 ± 1.2	< 0.001

Values represent the number of patients (percentage) or mean ± SD as appropriate. *p* values were obtained using the Pearson *χ*^2^ test and analyses of variance, as appropriate. IV, intravenous; SD, standard deviation; ICH, intracerebral hemorrhage; WBC, white blood cell; CRP, C-reactive protein; HDL, high-density lipoprotein; LDL, low-density lipoprotein; BUN, blood urea nitrogen; INR, international normalized ratio of prothrombin time.

**Figure 2:**
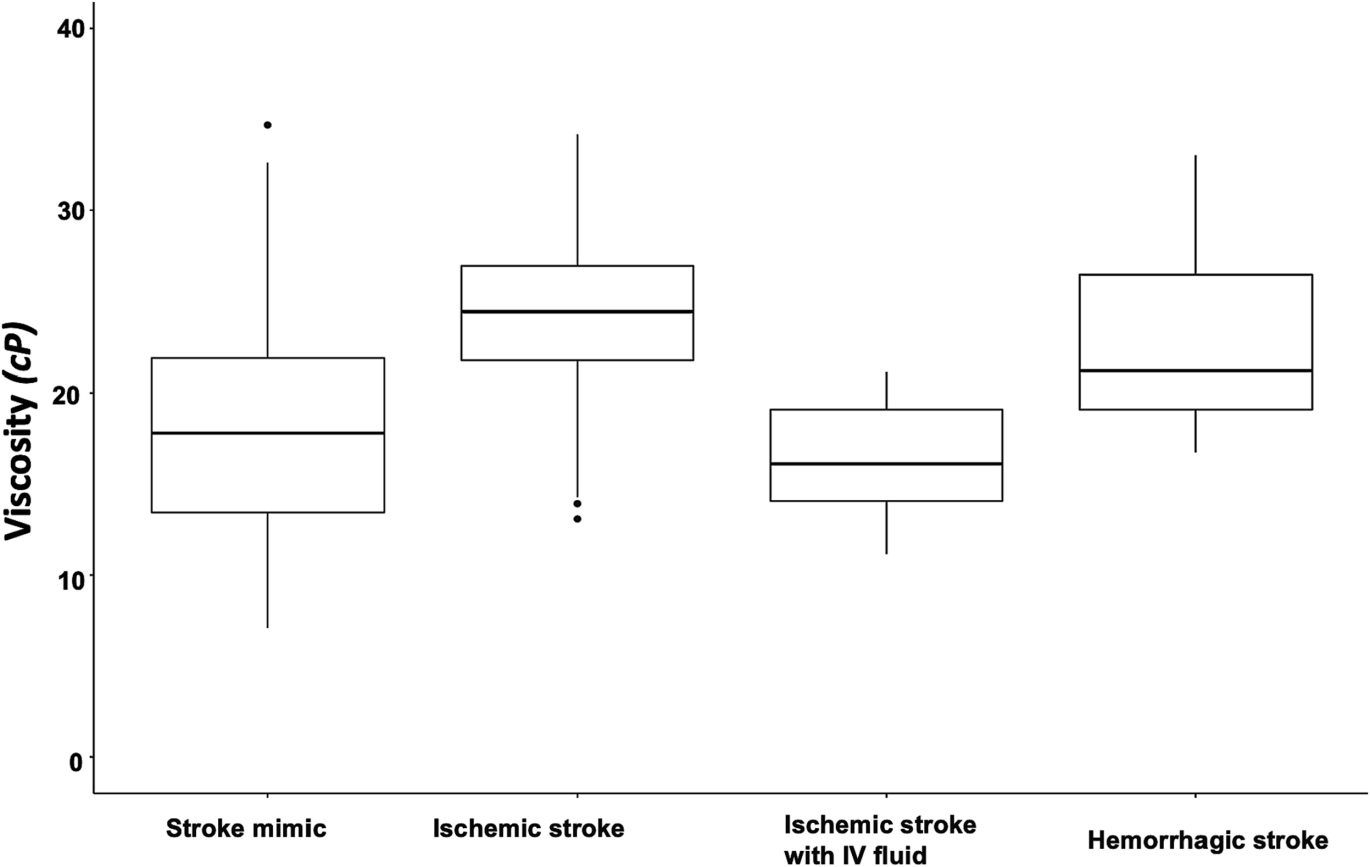
Box plot of blood viscosity according to disease groups. The graph shows the blood viscosity distribution in each disease group at the oscillatory frequency of 10 rad/sec (midline within the box for median values and the box itself for interquartile range). The blood viscosity value was 24.2 ± 4.9 *cP* in patients with ischemic stroke, which were significantly different from the stroke mimics (17.8 ± 6.5 *cP*), hemorrhagic stroke (23.2 ± 5.9 *cP*), and ischemic stroke with intravenous fluid infusion before the measurement (16.4 ± 3.3 *cP*).

**Figure 3:**
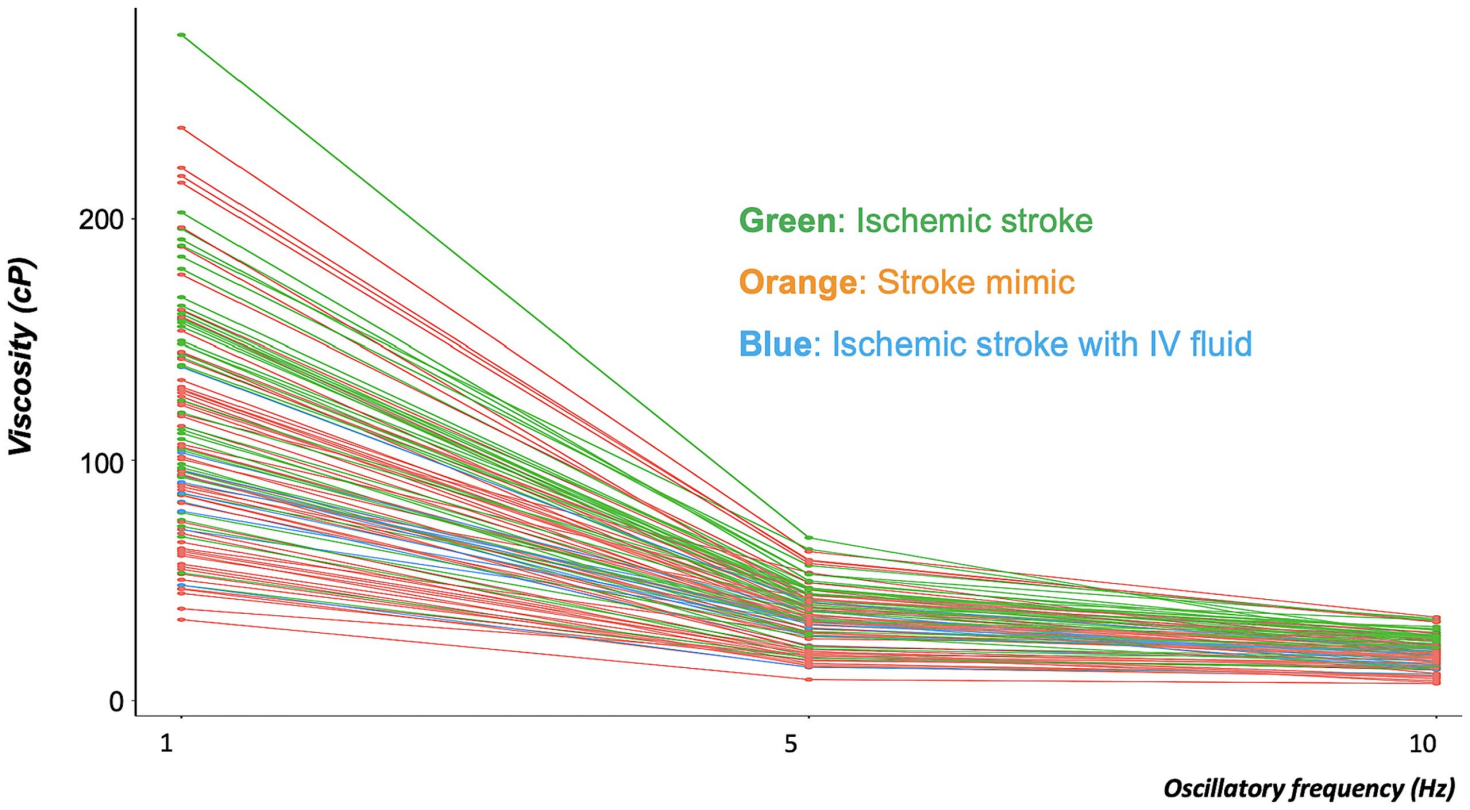
Distribution of blood viscosities in the ischemic stroke, ischemic stroke with IV fluid, and stroke mimic groups at three different oscillatory frequencies. The graph showed every inter-personal viscosity value (Y-axis) across the different oscillatory frequencies (X-axis). The line connected the individual measurement values. At each of the three oscillatory frequencies, blood viscosity was significantly higher in patients with ischemic stroke (green line) than in those with stroke mimic (orange line). Decreases in blood viscosity were observed in patients with ischemic stroke who had been treated with intravenous (IV) fluid (blue line).

**Figure 4:**
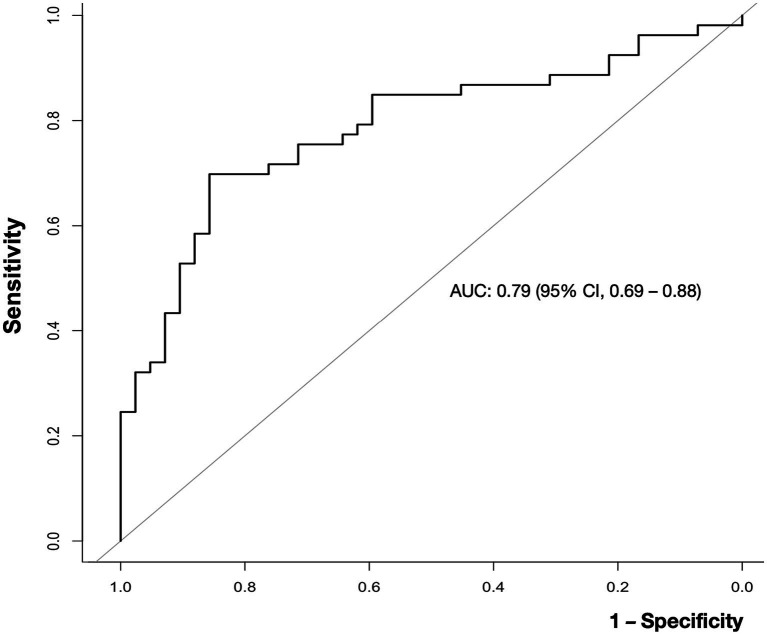
Receiver operating characteristic (ROC) curve for the ability of blood viscosity to differentiate ischemic stroke from other conditions. The area under the curve (AUC) value for blood viscosity differentiating ischemic stroke from non-stroke was 0.79 (95% confidence interval, 0.69–0.88).

Blood viscosity values were plotted with the disease groups (Figure 2). At an oscillatory frequency of 10 rad/sec, blood viscosity was 24.2 ± 4.9 *cP* in patients with ischemic stroke, which was significantly different from 17.8 ± 6.5 *cP* with stroke mimic, 23.2 ± 5.9 *cP* with hemorrhagic stroke (*P* by *ANOVA* < 0.001). The significant associations of higher viscosity values of ischemic stroke were consistently observed at lower oscillatory frequencies of 1 rad/sec (134.2 ± 46.3 vs. 102.4 ± 47.2 *cP*, *p* = 0.001) and 5 rad/sec (39.2 ± 11.5 vs. 30.4 ± 12.4 *cP*, *p* = 0.003, Figure 3 and Supplementary Table S4). Among patients diagnosed with ischemic stroke, blood viscosity at 10 rad/sec oscillatory frequency in those who received IV fluid before blood sampling was 16.4 ± 3.3 cP, which was similar to that observed in the stroke mimic group. The discrimination performance of blood viscosity measurements in the emergency setting exhibited good performance among the disease groups (AUROC = 0.79, Figure 4).

## 4 Discussion

To the best of our knowledge, this is the first study using a real-time, parallel plate viscosity measurement device to verify that patients with ischemic stroke have an increase in whole blood viscosity. Additionally, this property proposes that measuring blood viscosity may help distinguish ischemic stroke from other diseases. Although previous large-scale studies have highlighted the long-term effects of high blood viscosity on the risk of cardiovascular events (19), their measurements were limited by a high degree of noise and fluctuations in blood viscosity. From this perspective, our study strengthens the evidence regarding the effects of shear stress on endothelial injury or abrasion during thrombotic events (10).

Notably, even in patients with ischemic stroke, treatment with IV fluid was associated with a decrease in blood viscosity, which approached a level similar to that in the non-stroke group (median 23.5 vs. 14.7 *cP* at 10 rad/sec). This effect is related to the water content in normal saline or plasma solutions, which are Newtonian fluids with lowered viscosity. In addition to highlighting the sensitivity of blood viscosity measurements, this phenomenon may provide insight into the role of hemodilution or fluid treatment in patients with stroke, the benefits of which have remained controversial for some time (20, 21). Studies incorporating serial measurements of blood viscosity that account for hemodilution therapy may provide more accurate results regarding the clinical value of such treatment.

Considering the sensitivity of blood viscosity measurements to external factors, immediate measurements without anticoagulant use are indispensable for clinical utilization (15). The direct, on-site method used in the current study is advantageous because it directly measures blood viscosity while allowing for precise control of blood volume/additives. Moreover, POCT rheometer measurements have obtained an average of 2.3 min (median 2, IQR, 1–3) following a puncture, meaning that results were unlikely affected by spontaneous clotting, and the simplicity of the process means that no additional training is required.

On another point, this study only presented the significant associations of blood viscosity with hemoglobin levels and total cholesterol, not with white blood cell count or fibrinogen. This was because enrolled subjects had stroke or stroke mimic diseases, limited in hematologic diseases, so their laboratory value distributions were not wide enough to make the statistical significance (22). In addition, the high fibrinogen level in stroke mimics is thought to be due to a kind of infectious disease. Of note, lipoprotein, a major component of cholesterol, has an effect on the development and progression of atherosclerosis it can also infer it’s another biological role for the blood viscosity (10).

This study had several limitations, including its single-center design and small sample size. In addition, blood viscosity data in healthy controls was limited, and some patients had other risk factors or diseases that may impact the diagnostic utility of blood viscosity measurements. In another, further investigation of the association between blood viscosity and stroke subtypes had to be considered (14). Lastly, we still need to obtain serial blood viscosity measurements to investigate changes over time during admission, highlighting the need for more in-depth, long-term analyses.

In conclusion, this study observed the elevation of blood viscosity in ischemic stroke. The parallel-plate method enables us to measure the blood viscosity within a few minutes and would be helpful to distinguish the other stroke mimics.

## Data availability statement

The raw data supporting the conclusions of this article will be made available by the authors, without undue reservation.

## Ethics statement

The studies involving humans were approved by IRB of Seoul National University Bundang Hospital. The studies were conducted in accordance with the local legislation and institutional requirements. The participants provided their written informed consent to participate in this study.

## Author contributions

JiK: Conceptualization, Data curation, Formal analysis, Investigation, Methodology, Supervision, Visualization, Writing – original draft, Writing – review & editing. JO: Conceptualization, Funding acquisition, Investigation, Methodology, Resources, Visualization, Writing – review & editing. BK: Investigation, Resources, Supervision, Validation, Writing – review & editing. Juk: Data curation, Investigation, Resources, Validation, Writing – original draft. DK: Investigation, Methodology, Writing – review & editing. S-YY: Investigation, Methodology, Writing – original draft. M-KH: Investigation, Supervision, Writing – review & editing. H-JB: Investigation, Resources, Supervision, Writing – review & editing. IP: Conceptualization, Investigation, Resources, Writing – review & editing. JL: Investigation, Supervision, Validation, Writing – review & editing. YJ: Investigation, Supervision, Writing – review & editing. KA: Conceptualization, Investigation, Methodology, Writing – review & editing.
