# Supplementary material for: High blood viscosity in acute ischemic stroke
Source: Front Neurol. 2023 Nov 30;14:1320773. doi: 10.3389/fneur.2023.1320773 (PMC10723952; doi:10.3389/fneur.2023.1320773)
Supplement: Supplementary file 1 [file Data_Sheet_1.PDF]

# *Supplementary Material*

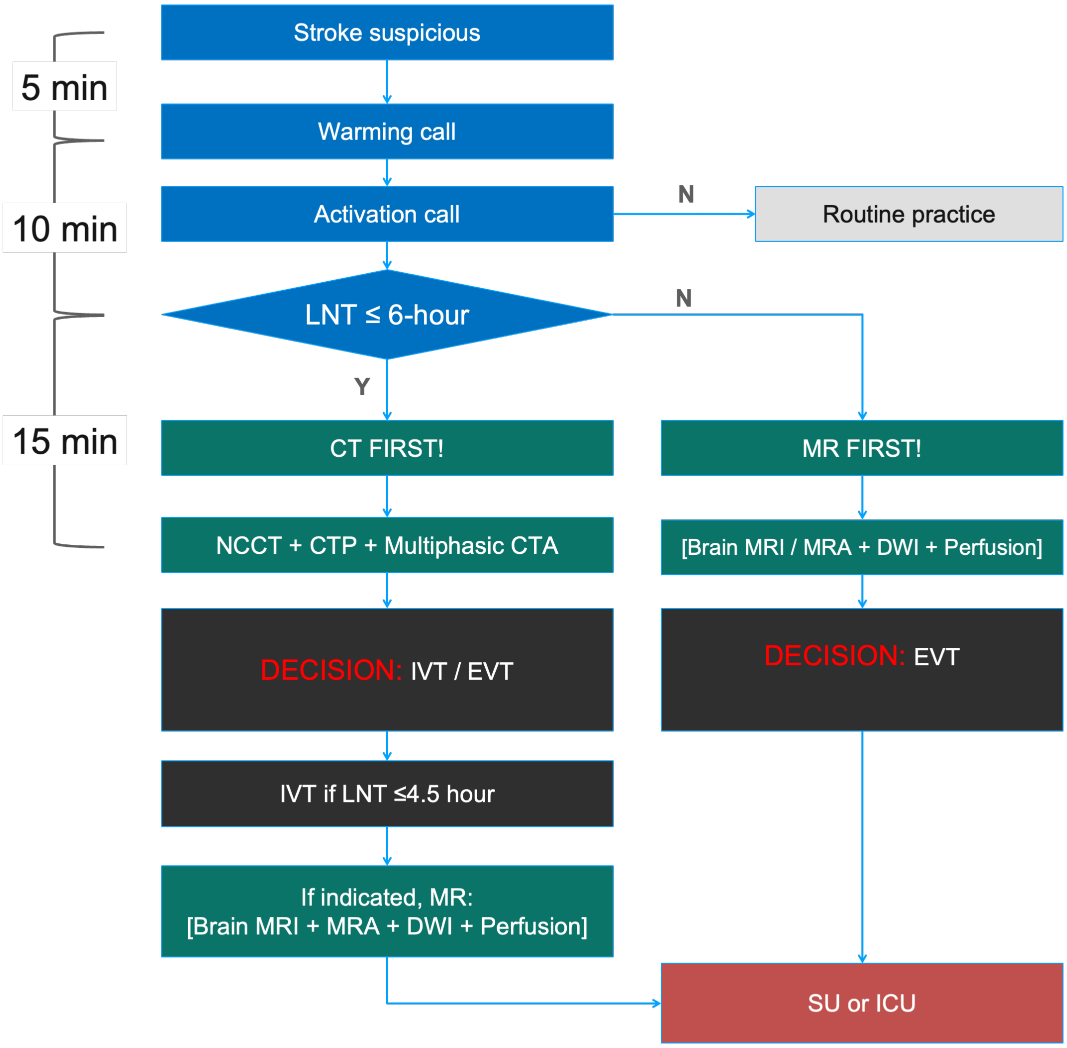


**Supplemental Figure 1**. Clinical pathway in cases of suspected stroke

This pathway is utilized when patients arrive within 24 hours of last normal time (LNT) with one of following signs or symptoms: 1) ipsilateral arm and/or leg weakness, 2) language disturbance, 3) visual field defect (or hemianopia), 4) altered mental status, 5) vertigo with hemiparesis or ataxia. CT, computed tomography; MRI, magnetic resonance imaging; NCCT, non-contrast CT; CTP, CT perfusion; CTA, CT angiography; MRA, MR angiography; DWI, diffusion-weighted imaging; IVT, intravenous thrombolysis; EVT, endovascular treatment; SU, stroke unit; ICU, intensive care unit.

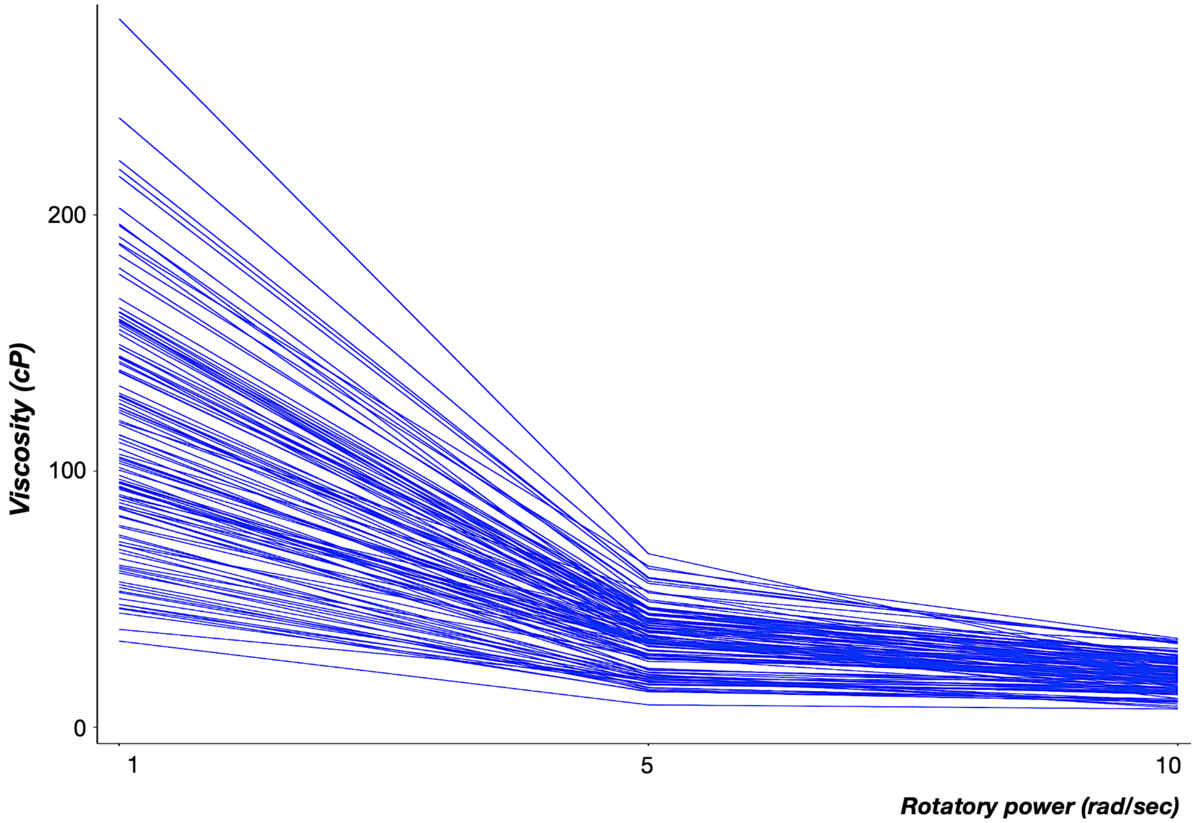


**Supplemental Figure 2**. Oscillatory frequency-dependent changes in blood viscosity.

Blood samples placed on the parallel plate were subjected to three oscillatory frequencies. Reactive torque in the blood was measured within 1 minute. Blood viscosity values at 1, 5, and 10 Hz were 115.4 ± 47.8 cP, 34.2 ± 12.2 cP, and 20.3 ± 6.5 cP, respectively, indicating that viscosity decreased as oscillatory frequency increased.

**Supplemental Table 1**. Comparison of parallel plate viscosity measurement methods to the capillary and cone-and-plate methods

| | **Parallel plate** | **Capillary** | **Cone-and-plate** |
|---|---|---|---|
| Whether to reflect internal viscosity | Good | Poor | Poor |
| Whether the blood clot reflects the viscosity | Good | Poor | Poor |
| Viscosity accuracy | Good | Neutral | Poor |
| Testing time | Short | Long | Long |
| Hygiene | Good | Good | Poor |

**Supplemental Table 2**. Correlation of blood viscosity with hematologic profiles at an oscillatory frequency of 10 *Hz*

| | Pearson coefficient ($\gamma$) | *Ps* |
|---|---|---|
| Age | -0.01 | 0.89 |
| Hemoglobin (*g/dL*) | 0.27 | 0.004 |
| Hematocrit (%) | 0.26 | 0.08 |
| Platelet ($10^3$/□L) | 0.10 | 0.30 |
| INR | -0.15 | 0.12 |
| D-dimer (*g/L*) | -0.20 | 0.18 |
| CRP (*mg/L*) | -0.18 | 0.07 |
| Glucose (*mg/dL*) | 0.07 | 0.44 |
| BUN (*mg/dL*) | -0.21 | 0.03 |
| Creatinine (*mg/dL*) | -0.16 | 0.09 |
| Total cholesterol (*mg/dL*) | 0.26 | 0.01 |
| HDL (*mg/dL*) | 0.09 | 0.35 |
| LDL (*mg/dL*) | 0.19 | 0.06 |
| Fibrinogen (*mg/dL*) | -0.06 | 0.54 |

Correlation coefficients (γ) and P values were obtained using Pearson correlation tests.

**Supplemental Table 3**. Detailed disease entities of stroke mimic group

| Disease entities | Number of patients (n = 53) |
|---|---|
| Dizziness | 8 (15.7%) |
| Brain tumor or metastasis | 4 (7.8%) |
| Syncope | 1 (2.0%) |
| Others not classified | 3 (5.9%) |
| Sequalae or recrudescence | 6 (11.8%) |
| Drug side effect or related symptom | 7 (13.7%) |
| Encephalopathy or CNS symptom of systemic illness | 16 (30.2%) |
| Peripheral neuropathy | 8 (15.7%) |

Values were the number of cases (percentage). CNS was abbreviated for the central nervous system. Others not classified were iatrogenic central retinal artery occlusion, subjective sensory symptom, and myositis.

**Supplemental Table 4**. Comparison of blood viscosity according to differently classified disease groups

A. Blood viscosity in patients with ischemic stroke, non-ischemic stroke, and ischemic stroke with IV fluid

| Oscillatory frequency | Non-ischemic stroke (n = 59) | Ischemic stroke (n = 42) | Ischemic stroke with IV fluid (n = 11) | P |
|---|---|---|---|---|
| 1 *Hz* | 106.9 ± 48.2 | 134.2 ± 46.3 | 89.4 ± 22.2 | 0.002 |
| 5 *Hz* | 31.2 ± 12.4 | 39.2 ± 11.5 | 31.1 ± 7.6 | 0.003 |
| 10 *Hz* | 18.3 ± 6.6 | 24.2 ± 4.9 | 16.4 ± 3.3 | < 0.001 |

B. Blood viscosity in patients with stroke mimic, ischemic stroke, ischemic stroke with IV fluid, and hemorrhagic stroke

| Oscillatory frequency | Stroke mimic (n = 53) | Ischemic stroke (n = 42) | Ischemic stroke with IV fluid (n = 11) | Hemorrhagic stroke (n = 6) | P |
|---|---|---|---|---|---|
| 1 *Hz* | 102.4 ± 47.2 | 134.2 ± 46.3 | 89.4 ± 22.2 | 146.2 ± 40.5 | 0.001 |
| 5 *Hz* | 30.4 ± 12.4 | 39.2 ± 11.5 | 31.1 ± 7.6 | 38.4 ± 10.0 | 0.003 |
| 10 *Hz* | 17.8 ± 6.5 | 24.2 ± 4.9 | 16.4 ± 3.3 | 23.2 ± 5.9 | < 0.001 |

Values are shown as mean ± SD. P values were estimated using analyses of variance for parametric data. Non-ischemic stroke was defined as stroke mimic or hemorrhagic stroke. Ischemic stroke with

IV fluid included those treated with IV fluid prior to blood sampling. IV, intravenous; SD, standard deviation.
